# Fixel based analysis of white matter alterations in early stage cerebral small vessel disease

**DOI:** 10.1038/s41598-022-05665-2

**Published:** 2022-01-28

**Authors:** Marvin Petersen, Benedikt M. Frey, Carola Mayer, Simone Kühn, Jürgen Gallinat, Uta Hanning, Jens Fiehler, Katrin Borof, Annika Jagodzinski, Christian Gerloff, Götz Thomalla, Bastian Cheng

**Affiliations:** 1 Department of Neurology, University Medical Center Hamburg-Eppendorf, Martinistraße 52, 20246 Hamburg, Germany; 2 Department of Psychiatry and Psychotherapy, University Medical Center Hamburg-Eppendorf, Hamburg, Germany; 3 Department of Diagnostic and Interventional Neuroradiology, University Medical Center Hamburg-Eppendorf, Hamburg, Germany; 4 Epidemiological Study Center, University Medical Center Hamburg-Eppendorf, Hamburg, Germany; 5 Department of General and Interventional Cardiology, University Heart and Vascular Center, Hamburg, Germany

**Keywords:** Neurovascular disorders, White matter disease

## Abstract

Cerebral small vessel disease (CSVD) is a common cause of morbidity and cognitive decline in the elderly population. However, characterizing the disease pathophysiology and its association with potential clinical sequelae in early stages is less well explored. We applied fixel-based analysis (FBA), a novel framework of investigating microstructural white matter integrity by diffusion-weighted imaging, to data of 921 participants of the Hamburg City Health Study, comprising middle-aged individuals with increased cerebrovascular risk in early stages of CSVD. In individuals in the highest quartile of white matter hyperintensity loads (n = 232, median age 63 years; IQR 15.3 years), FBA detected significantly reduced axonal density and increased atrophy of transcallosal fiber tracts, the bilateral superior longitudinal fasciculus, and corticospinal tracts compared to participants in the lowest quartile of white matter hyperintensities (n = 228, mean age 55 years; IQR 10 years). Analysis of all participants (N = 921) demonstrated a significant association between reduced fiber density and worse executive functions operationalized by the Trail Making Test. Findings were confirmed by complementary analysis of diffusion tensor metrics.

## Introduction

Age-related diseases pose a major burden on industrialized societies. The most common cerebrovascular disease in the elderly is cerebral small vessel disease (CSVD)—a condition conceived to arise from injury to the small perforating brain vessels. CSVD is defined by a heterogeneous composition of a clinical syndrome, histopathological features and imaging findings^1^. Initially deemed clinically insignificant, CSVD is now respected as a key contributor to vascular and Alzheimer’s dementia and triples the risk of stroke^2,3^. Cognitive decline, mood disturbances as well as gait impairment and urinary complaints are found to accompany CSVD as well^4–7^. The linkage of CSVD and its sequelae, however, remains poorly understood. Therefore, further characterization of structural brain changes induced by CSVD is needed to reveal its aetiopathogenesis.

Magnetic resonance imaging (MRI) is at the forefront of diagnostic modalities to investigate CSVD in-vivo. White matter hyperintensities of presumed vascular origin (WMH) are the most prominent manifestation and are considered a hallmark detectable in T2 weighted sequences (such as fluid-attenuated inversion recovery, FLAIR)^8^. MRI studies have furthermore revealed the dynamic nature of structural brain changes in CSVD: subtle, nonvisible T2-intensity changes are measurable in normal-appearing white matter (NAWM) before WMH occurrence^9^, followed by WMH growth with accelerating the progression of the disease^10,11^. However, conventional MRI approaches using T2-weighted sequences are insufficient to detect incipient changes of white matter integrity occurring at the microscopic level during the early stages of CSVD.

Diffusion-weighted imaging (DWI) on the other hand has shown to be sensitive to altered white matter integrity in CSVD^12^. Drawing its information solely from tissue water diffusion, modern DWI-based methods provide a manifold toolset for characterization of pathophysiological processes in neurological and psychiatric diseases: fibre tractography—i.e., the specific reconstruction of axonal trajectories from DWI information as streamlines—enables the approximation of the human brain network as a structural connectome. Structural connectomics demonstrated as insightful regarding the intricacies of the relationship between CSVD pathophysiology and symptoms^13,14^; analysis of DWI data by tensor imaging (DTI) is commonly applied to infer tissue status from diffusion properties of water molecules on a voxel scale generating metrics such as mean diffusivity (MD) and fractional anisotropy (FA). These metrics have been shown to quantify the extent of WMH and predict future WMH emergence in longitudinal investigations^15,16^. Notwithstanding, traditional DTI-derived metrics such as FA and MD are limited by their relatively low specificity of detecting microstructural changes, specifically in areas of complex fibre architecture present in the majority of white matter connections^17–19^.

Fixel-based analysis (FBA) is a novel framework that addresses the shortcomings of DTI and estimates microstructural changes of individual populations of specific white matter fiber bundles on a microscopic level. The term ‘fixel’ was introduced to describe a *fi*bre population within a specific vo*xel*. FBA generates three metrics, fibre density (FD), fibre-bundle cross-section (FC) and their combined effect (FDC). These metrics are considered to capture changes of intraaxonal volumes of axons aligning with a specific fibre population (FD) and macroscopic cross-sectional size of individual fibre bundles (FC). They reflect on pathophysiological processes such as fibre loss or atrophy and thus allow for direct assessment of microstructural changes on a subvoxel level^20,21^.

We therefore applied FBA to investigate micro- and macrostructural white matter changes in participants of the Hamburg City Health Study (HCHS) a population-based study including individuals with increased risk for cerebrovascular diseases. We hypothesized that in this population, early-stage CSVD leads to white matter alterations detectable by FBA metrics. As cognitive function is commonly acknowledged as affected in cerebral small vessel disease we furthermore aimed to demonstrate associations between FBA metrics and results of the Trail Making Test which is a neuropsychological test considered to operationalize frontal-executive cognitive function by capturing set shifting and psychomotor speed performance^22,23^. We complemented our study with an analysis of established DTI-based metrics for qualitative comparisons.

## Methods

### Study population—the Hamburg City health study

We investigated clinical and imaging data from participants of the Hamburg City Health Study (HCHS), a single-center prospective, epidemiologic cohort study with emphasis on imaging to improve the identification of individuals at risk for major chronic diseases and to improve early diagnosis and survival. A detailed description of the overall study design has been published separately^24^. In brief, 45,000 citizens of the city of Hamburg, Germany, between 45 and 74 years are invited to an extensive baseline evaluation. A subgroup of participants with increased cerebrovascular risk factors as defined by a Framingham Stroke Risk Score > 8 undergoes standardized MRI brain imaging^25^. Of this subgroup, we analyzed data from the first 1,000 participants with available brain MRI. Neuropsychological assessment is conducted in all participants of the HCHS. For our study, we probed for an association of structural white matter changes and performance in frontal-executive cognitive functions known to be relevantly impaired in patients with CSVD^23^. Therefore, test scores from the Trail Making Test A (TMT-A) and B (TMT-B) were chosen. During component A, participants were asked to connect dots with numbers from 1–25 in an incremental order. Its results are thought to reflect psychomotor speed, visuospatial search and target-directed motor tracking. Component B complicates that endeavour by requiring the participant to alternate between numbers and letters adhering to the numerical and respectively alphabetical order. Performance in component B is considered to rely more on higher-order cognitive function as it is correlated with results of commonly used tests of executive function^26^. The difference between both measures (TMT-B—TMT-A) here referred to as TMT-delta was assessed as well to isolate the contribution of executive function to the test results^27^.

### Ethics approval and consent

The local ethics committee of the Landesärztekammer Hamburg (State of Hamburg Chamber of Medical Practitioners, PV5131) approved the study and written informed consent was obtained from all participants. Good Clinical Practice (GCP), Good Epidemiological Practice (GEP) and the Declaration of Helsinki were the ethical guidelines that governed the conduct of the study^28^.

### MRI acquisition

Images were acquired using a 3-T Siemens Skyra MRI scanner (Siemens, Erlangen, Germany). Measurements were performed adapting a protocol as described previously^29^. In detail, for single-shell diffusion-weighted imaging (DWI), 75 axial slices were obtained covering the whole brain with gradients (b = 1000 s/mm^2^) applied along 64 noncollinear directions with the following sequence parameters: repetition time (TR) = 8500 ms, echo time (TE) = 75 ms, slice thickness (ST) = 2 mm, in-plane resolution (IPR) = 2 × 2 mm, anterior–posterior phase-encoding direction, 1 b0 volume. For 3D T1-weighted anatomical images, rapid acquisition gradient-echo sequence (MPRAGE) was used with the following sequence parameters: TR = 2500 ms, TE = 2.12 ms, 256 axial slices, ST = 0.94 mm, and IPR = 0.83 × 0.83 mm. 3D T2-weighted fluid-attenuated inversion recovery (FLAIR) images were measured with the following sequence parameters: TR = 4700 ms, TE = 392 ms, 192 axial slices, ST = 0.9 mm, and IPR = 0.75 × 0.75 mm.

### Data preprocessing

Multimodal neuroimaging data has been analyzed using MRtrix 3.0 (http://www.mrtrix.org), Advanced Normalization Tools (ANTs, https://github.com/ANTsX/ANTs), the FMRIB Software Library 5.0.10 (FSL, https://fsl.fmrib.ox.ac.uk) and TractSeg (https://github.com/MIC-DKFZ/TractSeg)^30–32^. Preprocessing of diffusion-weighted images (DWI) comprised denoising^33^, deringing^34^, eddy current distortion^35^ and bias correction^36^. Head motion was corrected via FSL eddy’s builtin replacement of outlier slices (–repol). ANTs’ SyN was utilized to accomplish susceptibility distortion correction registration: the DWI was non-linearly registered on a T1-weighted image which was histogram-matched to the corresponding b0 beforehand^37^. Total brain volume was derived from the brain mask acquired by applying ANTs brain extraction to the T1-weighted image.

### White matter hyperintensity segmentation

We segmented WMH using the Brain Intensity AbNormality Classification Algorithm (BIANCA) implemented in FSL^38^. The training dataset consisted of masks of 100 participants which were obtained by selecting only the voxels that were identified as WMH by two raters (MP, CM) independently via manual segmentation. Mean Dice Similarity Index between segmentations of both raters was 0.63. Resulting segmentation masks underwent visual quality control. WMH loads were calculated as the percentage of WMH volume in total brain volume.

### Computation of fixel metrics

**Figure 1:**
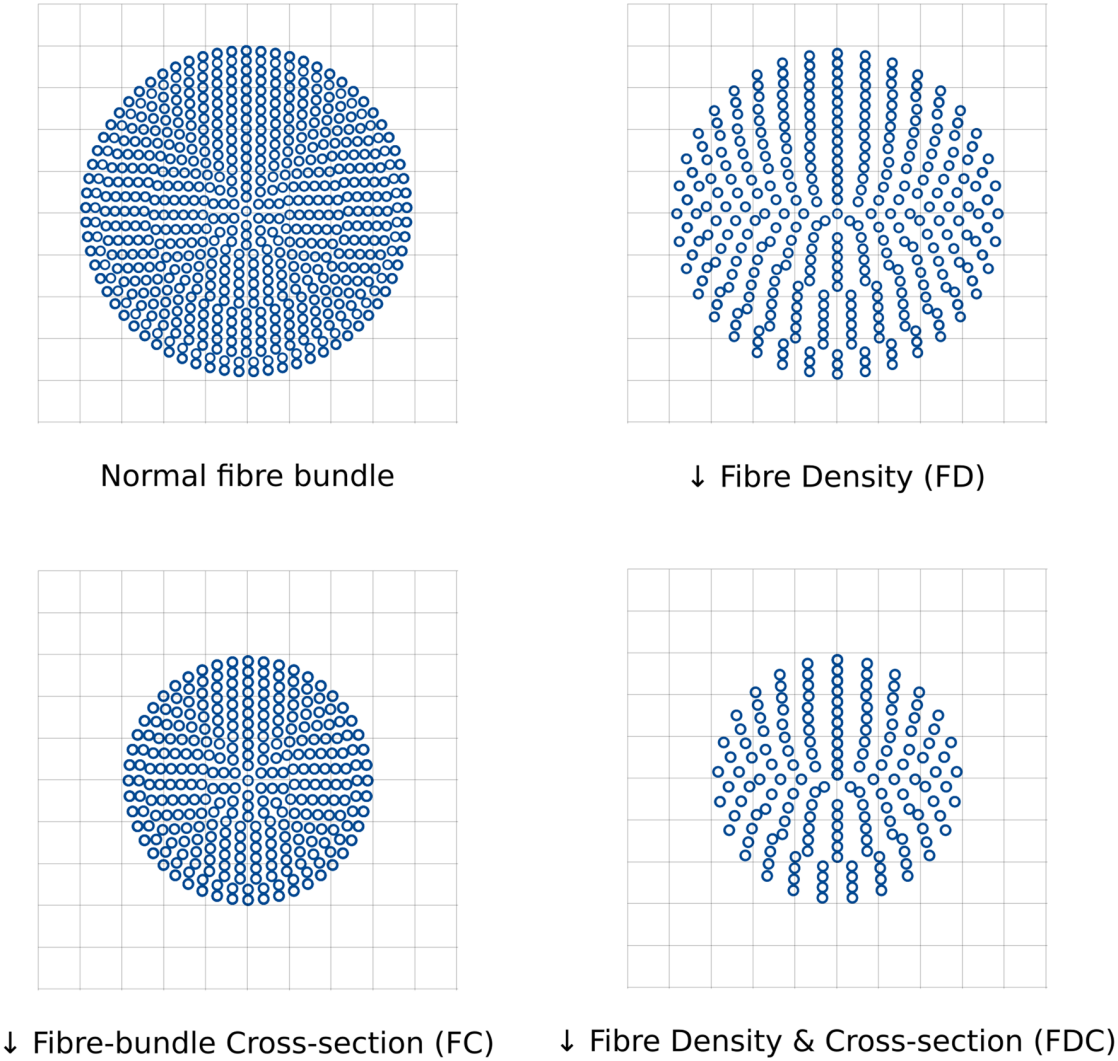
HYPERLINK "sps:id::fig1||locator::gr1||MediaObject::0" Fixel metrics. Diagrammatic illustrations for all fixel metrics investigated are shown. Starting from a healthy fibre bundle differing structural changes can appear that are reflected by specific metrics. A loss in intraaxonal volume in a voxel is indicated by a reduction in fibre density. A reduced fibre-bundle cross-section reflects macrostructural—i.e., above voxel level—tract diameter changes in the plane orthogonal to the fixel surveyed. Fibre density and cross-section represents the product of FD and FC and is thus sensitive to combined structural changes. This figure is modification from Fig. 1 of Raffelt et al. 2017 and was produced using inkscape (https://inkscape.org/).

Fixel metrics were computed and interpreted adhering to the recommended methodology^21^. DWI was resampled to an isotropic voxel size of 1.3. Response functions were derived from DWI and averaged over the whole sample. Subsequently, fibre-orientation distributions (FOD) were computed using single-shell 3-tissue constrained spherical deconvolution^39,40^. For spatial correspondence, an unbiased FOD group template was computed from 20 subjects of each group^41^. All subjects’ FOD images were non-linearly registered to the group template and fixel-based metrics were derived for each subject. Figure 1 illustrates the interpretation of the derived fixel metrics:

1. Fibre density (FD) is a subvoxel metric and represents the integral of the underlying FOD. It reflects the intra-axonal volume of fibre tracts matching the fixel direction. Of note, our b-value of b = 1000 s/mm^2^ is smaller than the recommendation for quantifying FD^21^. However, our approach follows previous studies yielding plausible results based on single-shell data with b = 1000 s/mm^2^^42,43^.
2. Fibre-bundle cross-section (FC) measures differences in fibre-bundle diameters between a subject’s FOD and the FOD template. It is defined as the degree of distortion in the perpendicular plane of a fixel’s orientation that is necessary to register the subject FOD to the template FOD. All results reported refer to the logarithmic FC.
3. Fibre density and cross-section (FDC) is the combined measure of FD and FC and allows for simultaneously assessing microscopic and macroscopic alterations of white matter tracts.

Probabilistic tractography of 20 million streamlines was performed on the group template followed by spherical-deconvolution informed filtering of tractograms resulting in a whole-brain template tractogram comprising 2 million streamlines^44^. WMH masks were warped onto the population template by utilizing linear and nonlinear information of the registration path from FLAIR to template space.

### Computation of DTI metrics

TBSS was performed using FSL and MRtrix3 adhering to the recommended pipeline (https://fsl.fmrib.ox.ac.uk/fsl/fslwiki/TBSS/UserGuide) with the variation of warping to the group template instead of standard space^45^. DTI metric maps of fractional anisotropy and mean diffusivity were derived applying dtifit as implemented in FSL to preprocessed DWI in individual space. The maps were registered to template space applying warps generated during the registration of FODs to the group template as described beforehand. A TBSS skeleton was reconstructed from the mean image of warped FA maps enabling statistical analysis on a geometrically matched set of white matter voxels. Warped FA and MD maps were projected onto the FA skeleton.

### Statistical analysis

For analysis of associations between FBA and DTI metrics with WMH burden, the sample was split into quartiles of WMH volumes and only data from participants in the first quartile (lowest WMH volumes, hereinafter referred to as WMH-Q1) and last quartile (highest WMH volumes, referred to as WMH-Q4) were chosen. For analysis of associations between FBA and DTI metrics with TMT scores, the complete sample was used. Differences in sample characteristics were investigated with a t-test for normally distributed continuous data and χ2 for categorical data. The resulting p-values were corrected for multiple comparisons according to Bonferroni.

### Whole-brain fixel-based analysis and tract-based spatial statistics

A whole-brain FBA was applied to identify differences of FBA metrics comparing groups with lowest and highest WMH volumes (WMH-Q1 vs. WMH-Q4) using fixel-wise general linear models (GLM) adjusted for age, sex and total brain volume. Connectivity-based fixel enhancement (CFE) was performed to achieve smoothing and statistical inference based on the template tractogram^46^. Resulting p-values were family-wise error corrected with 5000 permutations^47^. For DTI metrics, tract-based spatial statistics was conducted for comparison across groups (WMH-Q1 vs. WMH-Q4) using the same covariates as above and applying threshold-free cluster enhancement (TFCE) with 5000 permutations^48^.

### Tract of interest analysis

For anatomical localization of FBA metrics changes, TractSeg, a deep learning-based tool for white matter tract segmentation, was used to obtain binary masks and bilateral tractograms of 27 commonly investigated white matter fibre bundles: arcuate fasciculus (AF), anterior thalamic radiation (ATR), cingulum (CG), corticospinal tract (CST), inferior fronto-occipital fasciculus (IFO), inferior longitudinal fasciculus (ILF), superior longitudinal fasciculus (SLF I-III) uncinate fasciculus (UF) and the corpus callosum segments 1–7 (CC 1–7)^32,49^. FBA metrics were surveyed for and averaged over every tract-specific fixel mask. In addition, we performed a tract-specific comparison of WMH lesion loads. Linear mixed-effects models were used for tract-specific statistical comparison of metrics across groups. Age, sex and total brain volume were added as fixed effects and subject ID as a random effect to the models involving fixel metric comparison and p-values were Bonferroni corrected for multiple comparisons. As FDC is the most comprehensive and robust metric in FBA, we only consider the FDC for the tract of interest analysis in the main manuscript^21^. Complete results of all metrics are reported in the supplementary materials (supplementary Fig. 3–4 and Tables 1–3). Since our main focus is the investigation of FBA metrics, this analysis was not repeated for DTI metrics.

### Association with cognitive test results

We assessed associations with FBA and DTI metrics with executive cognitive functions by applying whole-brain fixel-wise and TBSS voxel-wise GLM in data from all participants included in this study. Fixel metrics (FC, FD, FDC) or DTI metrics (FA, MD) were used as the independent variables and TMT-A, TMT-B as well as TMT-delta as the dependent variables, resulting in 20 separate analyses. Positive and negative contrasts were tested. Age, sex, total brain volume and years of education were added as covariates and family-wise error correction was performed using CFE or TFCE analogous to whole-brain FBA and TBSS described above.

If not stated otherwise, p-values reported in this work are corrected for multiple comparisons and were deemed statistically significant if remaining lower than 0.05. Statistical computations were performed in R 3.6.1 and Python 3.7. Utilised packages were ggplot2, lmerTest and pandas^50–52^.

## Results

### Sample characteristics

Data from 1000 participants of HCHS were included in this analysis. Quality assessment ruled out 79 subjects due to missing data (n = 21), insufficient imaging quality (n = 40), or failed image processing (n = 18). In total, data from n = 921 participants were available for analysis. Table 1 provides an overview of demographic and imaging data. Median age was 63 years (IQR = 15.25), 47.4% of participants were female, median total brain volume was 1459.5 ml (IQR = 209.7) and median WMH load 0.117% (IQR = 0.220). For analysis of associations with the TMT, data from n = 783 (TMT-A) and n = 776 (TMT-B) participants were available. Based on the volume of WMH, 228 subjects were grouped into the first quartile (lowest WMH volumes, WMH-Q1) and 232 into the last quartile (highest WMH volumes, WMH-Q4). Both groups were well matched regarding sex (χ2 = 0.415, p = 1) as well as total brain volume (t = 1.52, p = 1) but significantly differed in terms of age (t = − 19.21, p < 0.001), years of education (t = 5.72, p < 0.001) and WMH load (t = − 21.63, p < 0.001). Regarding cerebrovascular risk factors WMH-Q4 encompassed significantly more participants with arterial hypertension (χ2 = 23.25, p < 0.001).

**Table 1:** Sample characteristics and image analysis results.

	All	WMH-Q1	WMH-Q4	Statistic
	n = 921	n = 228	n = 232	
Age, years (IQR)	64.0 (14.0)	56.0 (10.0)	70.5 (7.0)	t = − 19.21
				p < 0.001
Females, n (%)	419 (45.5)	112 (49.1)	106 (45.7)	χ2 = 0.415
				p = 1
Brain volume, ml (IQR)	1457.532 (197.5)	1474.190 (199.5)	1445.551 (211.9)	t = 1.52
				p = 1
WMH load, % (IQR)	0.044 (0.101)	0.007 (0.008)	0.227 (0.188)	t = − 21.63
				p < 0.001
Current smokers, n (%)	144 (15.6)	38 (16.7)	38 (16.4)	χ2 = 0.002
				p = 1
Diabetes *, n (%)	74 (8.0)	11 (4.8)	29 (12.5)	χ2 = 6.788
				p = 1
Hypertension **, n (%)	631 (68.5)	129 (56.6)	186 (80.2)	χ2 = 23.25
				p < 0.001
BMI (IQR)	25.75 (5.54)	25.75 (5.54)	26.69 (5.57)	t = − 0.799
				p = 1
Years of education (IQR)	13 (4)	14.5 (4)	13 (3)	t = 5.72
				p < 0.001
MMSE (IQR)	28 (2)	29 (1)	28 (2)	t = 3.622
				p = 1
TMT-A, seconds (IQR)	36 (17)	33 (14)	42 (18.5)	t = − 6.244
				p < 0.001
TMT-B, seconds (IQR)	79 (37)	68 (30)	88 (43)	t = − 6.015
				p < 0.001
TMT-delta, seconds (IQR)	40 (30)	37 (22)	45 (34)	t = − 4.195
				p < 0.01

Displayed p-values were Bonferroni-corrected for multiple comparisons.

*WMH* White Matter Hyperintensities, *WMH-Q1* individuals in the lowest quartile of WMH load, *WMH-Q4* individuals in the highest quartile of WMH load, *MMSE* mini-mental state examination, *TMT-A* trail making test a, *TMT-B* trail making test b, *TMT-delta* difference of TMT-B and TMT-A.

*Presence of diabetes was defined as a blood glucose level > 126 mg/dl or a self-reported prevalence of diabetes.

**Presence of hypertension was defined as blood pressure ≥ 140/90 mm/Hg, hypertensive medication intake or self-reported prevalence of hypertension.

### Analysis of group differences in FBA and DTI metrics between low and high WMH burden

**Figure 2:**
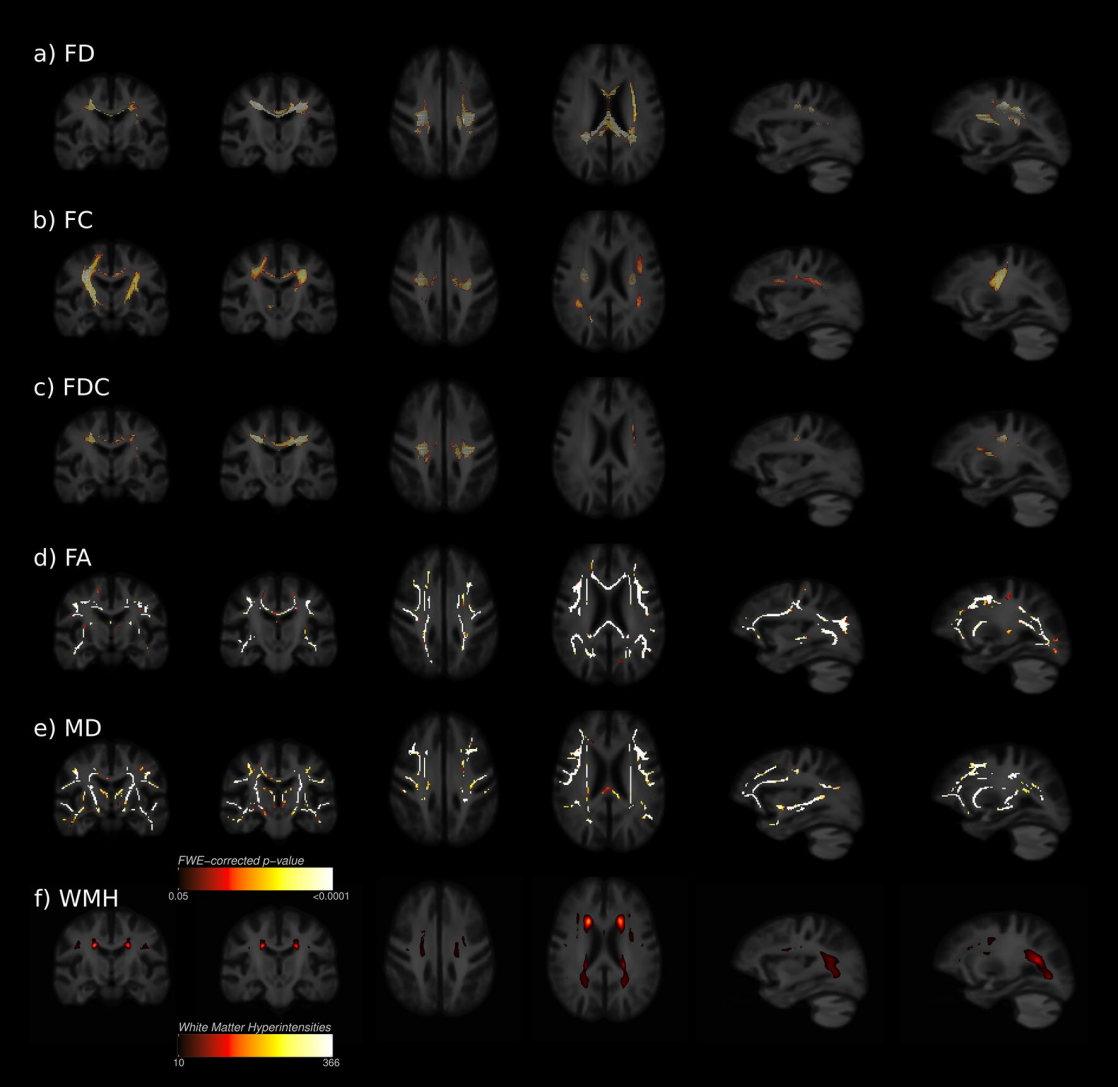
Whole-brain fixel-based analysis and TBSS. Fixels or voxels exhibiting significantly differing diffusion metrics (FWE-corrected p < 0.05) between groups are shown on the population template and colour-coded according to their significance level with the same colorbar applying to both analyses. Coronal, axial as well as left hemispheric sagittal slices are displayed. (**a**–**c**) whole-brain FBA results. Tract correspondences mentioned below were identified using tract segmentations acquired from TractSeg. Regions that significantly differed in terms of FD and FDC corresponded to the corpus callosum and SLF. Regarding the FC the CST and SLF were highlighted. (**d**,**e**) TBSS results. Significant TBSS skeleton voxels of FA and MD were widely distributed over the whole skeleton including frontal, temporal, parietal and occipital white matter. (**f**) WMH heatmap. *AF* arcuate fasciculus, *FA* fractional anisotropy, *FBA* fixel-based analysis, *FD* fibre density, *FC* fibre-bundle cross-section, *FDC* Fibre density and cross-section, *MD* mean diffusivity, *SLF* superior longitudinal fasciculus, *TMT-delta* difference of trail making test b and a, *WMH* white matter hyperintensities. Figure was produced using inkscape and mrview from MRtrix3 (https://www.mrtrix.org/).

Between participants with high (WMH-Q4) and low WMH (WMH-Q1) burden, whole-brain FBA identified regions with altered FD, FC and FDC in the cerebral white matter as visualized in Fig. 2a–c. Lower FD was shown in WMH-Q4 located at the corpus callosum (CC), preferentially the genu and body of the CC and its peripheral trajectory. A small area containing crossing fibres in the left hemispheric deep white matter (corresponding with the superior longitudinal fasciculus) showed significantly lower FD as well. Macrostructural changes were detected as a significantly lower FC in WMH-Q4 participants along the corticospinal tracts of both sides. Furthermore, regions comprising the body of corpus callosum and the SLF showed a significantly lower FC in WMH-Q4. Significant differences in FDC were mostly found in regions where alterations in FD and FC overlapped, namely the CC and its peripheral trajectories. Figure 2f illustrates that although altered FBA metrics were detected in brain areas with frequent WMH occurrence, significant changes were also detected beyond brain areas of manifest WMH in white matter tracts such as the SLF, corpus callosum or cortico-spinal tract. For DTI metrics, TBSS results demonstrate that WMH-Q4 participants showed significant decreases of FA and significant increases of MD in a pattern covering large sections of the white matter skeleton including callosal areas, the centrum semiovale and deep white matter of the frontal, temporal, parietal and occipital lobes (Fig. 2d,e).

### Anatomical localization of differences in FBA metrics

**Figure 3:**
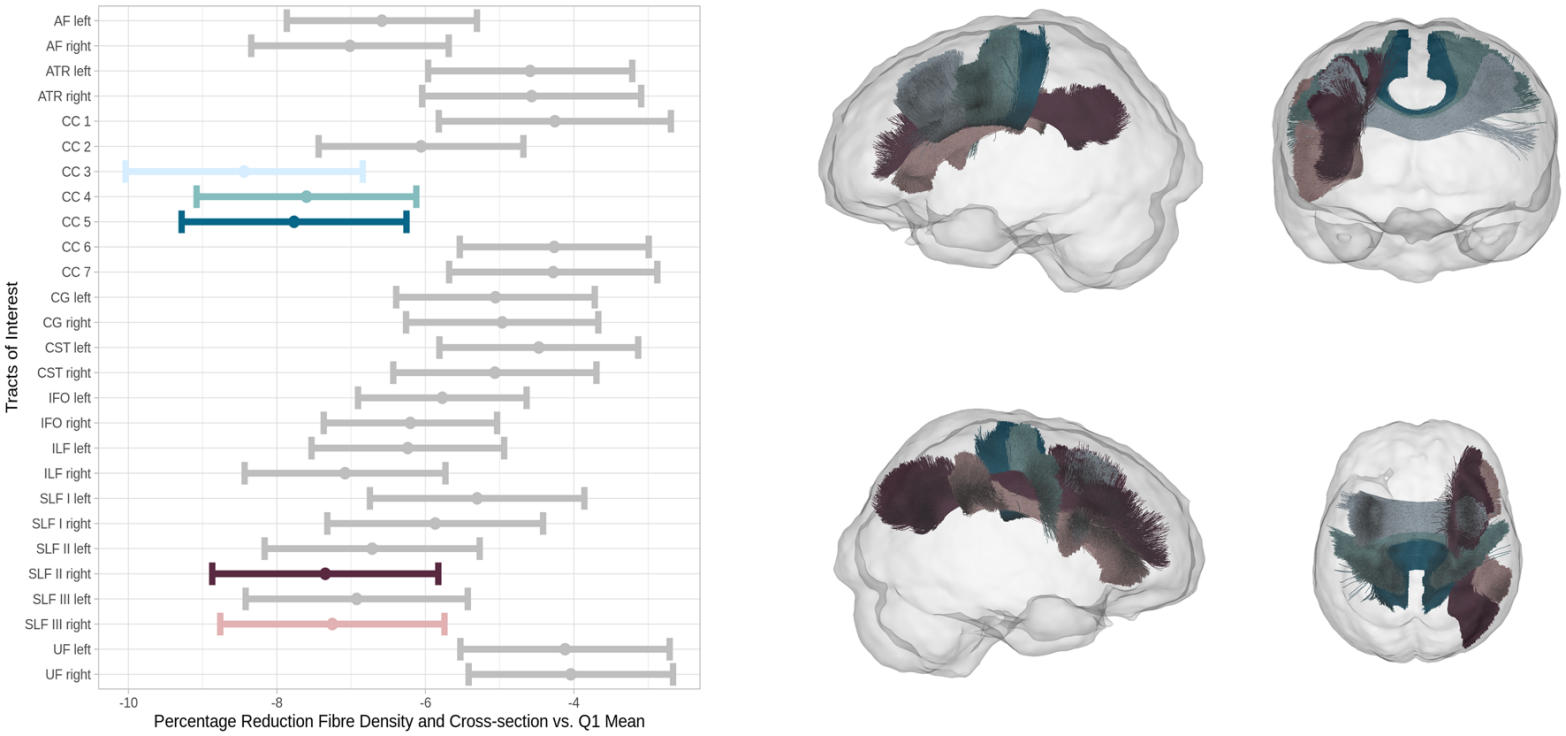
Tract of interest analysis. Left: error plots showing mean fibre density and cross-section percentage changes of the group of higher CSVD burden (WMH-Q4) with respect to the mean of the lower CSVD burden group (WMH-Q1, at x = 0) and corresponding 95% confidence intervals. Colour denotes significant tracts. Right: significantly differing tracts coloured corresponding to the left panel. Tracts are visualised from different perspectives: looking from left (upper left), from anterior (upper right), from right (lower left) and from superior (lower right). *AF* arcuate fasciculus, *ATR* anterior thalamic radiation, *CC* corpus callosum, *CG* cingulum, *CST* corticospinal tract, *IFO* inferior frontooccipital fasciculus, *ILF* inferior longitudinal fasciculus, *SLF* superior longitudinal fasciculus, *UF* uncinate fasciculus. Figure was produced using ggplot2^50^.

**Figure 4:**
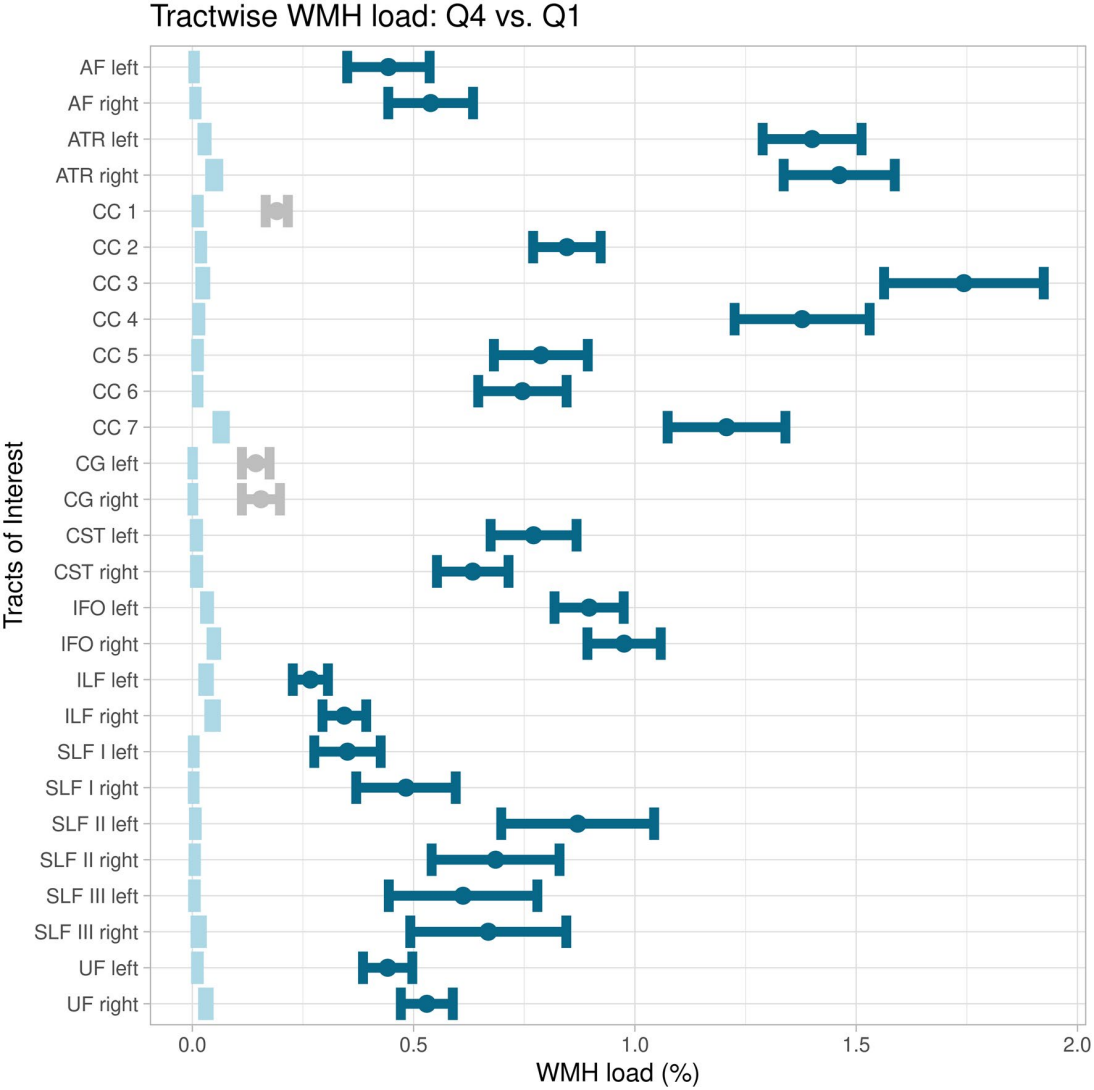
Tract-wise comparison of white matter hyperintensity loads. Mean WMH loads are depicted as dots and 95% confidence intervals as corresponding error bars. Intervals of the lower CSVD burden group (WMH-Q1) are coloured light blue. If significantly differing in terms of WMH load, intervals of the higher CSVD burden group (WMH-Q4) are coloured in dark blue and if not grey. *AF* arcuate fasciculus, *ATR* anterior thalamic radiation, *CC* corpus callosum, *CG* cingulum, *CST* corticospinal tract, *IFO* inferior frontooccipital fasciculus, *ILF* inferior longitudinal fasciculus, *SLF* superior longitudinal fasciculus, *UF* uncinate fasciculus, *WMH* white matter hyperintensity. Figure was produced using ggplot2^50^.

The tract of interest analysis revealed significantly lower FDC of participants with higher WMH load (WMH-Q4) in the rostral body (p < 0.001, CC 3) as well as the anterior (p = 0.007, CC 4) and posterior (p = 0.003, CC 5) midbody of the corpus callosum and in the subregions CC 2 (p = 0.021) and CC 3 (p = 0.031) of the right SLF (Fig. 3). Figure 4 shows the comparison of tract-wise WMH load. Except for the rostrum of the corpus callosum (CC 1) and both cingulate gyri (CG), all tracts investigated showed significantly differing WMH loads in WMH-Q4 participants.

### Association of FBA and DTI metrics with cognitive test results

**Figure 5:**
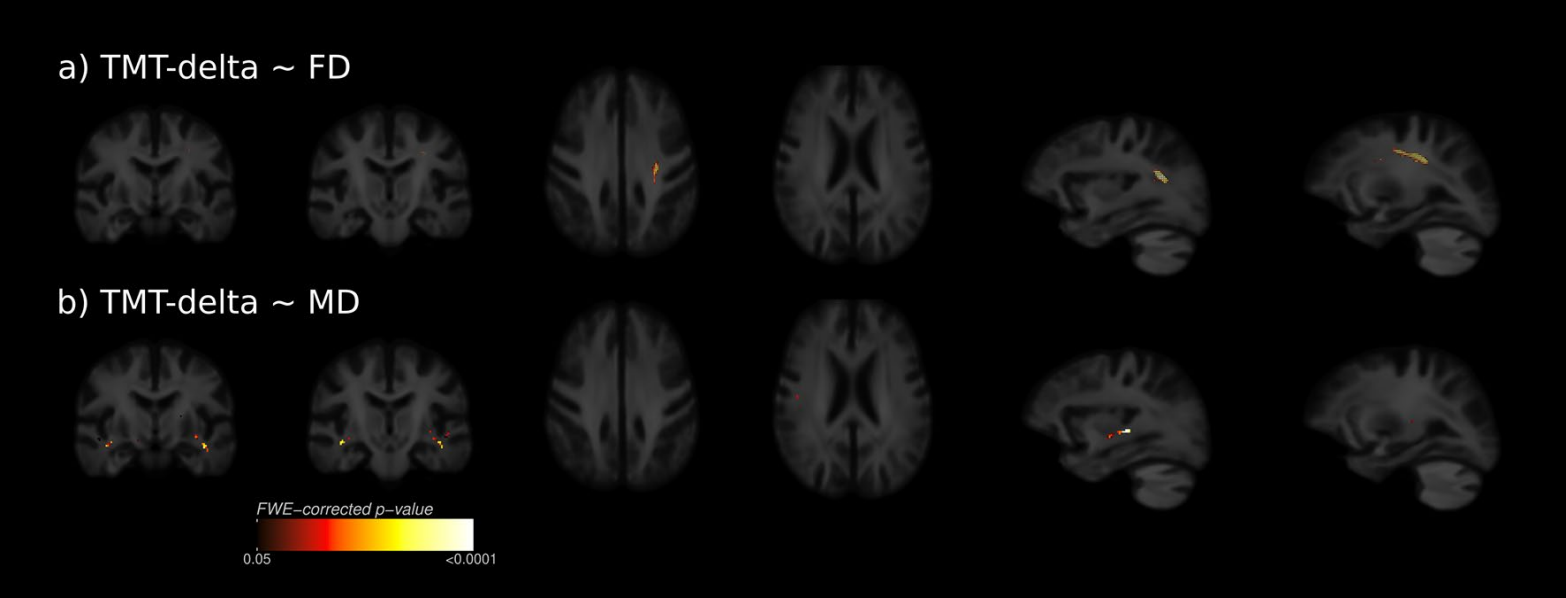
Whole-brain clinical fixel-based analysis and TBSS. Fixels and voxels that show a significant linear relationship (FWE-corrected p < 0.05) between metrics and the difference of TMT-B and TMT-A (TMT-delta) are visualised on the population template. (**a**) the SLF showed a significant linear relationship between FD and TMT-delta. (**b**) the MD was significantly correlated with TMT-delta bilaterally in temporal areas traversed by the AF and ILF. *AF* arcuate fasciculus, *FD* fibre density, *FC* fibre-bundle cross-section, *FDC* Fibre density and cross-section, *ILF* inferior longitudinal fasciculus, *MD* mean diffusivity, *TMT-delta* difference of trail making test b and a. Figure was produced using inkscape and mrview from MRtrix3 (https://www.mrtrix.org/).

Results from whole-brain FBA and DTI TBSS regarding TMT-delta are shown in Fig. 5. We detected a significant negative association between the FD and the TMT-delta in the left superior longitudinal fasciculus. MD showed bilaterally a positive association with TMT-delta in deep white matter voxels of the temporal lobe which corresponded with the arcuate fasciculus and inferior longitudinal fasciculus. This indicates that low fibre density and an increased magnitude of water diffusivity were associated with longer processing times determined by the TMT-delta. No significant associations between FDC, FC and FA and the TMT-delta were detected. Significance maps regarding the TMT-A and -B are illustrated in the supplementary materials (supplementary Fig. 1–2). Here, the pattern of significant fixel distributions was of a larger spatial extent regarding the TMT-B.

## Discussion

We report on white matter alterations assessed by FBA in a large population-based sample of individuals at risk of cerebrovascular disease and in early stages of CSVD. Our study has two main findings: first, higher extent of WMH was accompanied by changes of FBA metrics capturing white matter alterations on a microscopic and macroscopic level at the corpus callosum, superior longitudinal fasciculus and corticospinal tract, whereas DTI metrics showed a less specific and more general pattern of changes. Second, FBA and DTI metrics were associated with lower performance in the TMT indicating impaired frontal-executive cognitive functions. These results promote FBA metrics as a novel imaging marker of microstructural white matter alterations linked to clinical behavior in early stages of CSVD.

Conceptually, the application of FBA offers several advantages compared to DTI-based methods. The fibre tract-specific model provides measures more directly interpretable regarding disturbed structural connectivity as compared to voxel-average measures such as FA. FBA promotes statistical testing beyond the confinement of a white matter ‘skeleton’ of classical tract-based statistics, which can be limited by reduced detection accuracy compared to whole-brain analysis^53,54^. Furthermore, FBA is helpful to guard against potential misleading findings in brain areas with complex crossing fibre architecture, that are known to result in erroneously unchanged or increased values of voxel-average DTI metrics such as FA as demonstrated recently in a comparative investigation of patients with Alzheimer’s disease^55,56^.

In our population-based sample of individuals with an overall low amount of WMH (median WMH load in WMH-Q1 = 0.007%), we identified regions of reduced FD, indicating microstructural fibre-specific axonal loss, reduced FC, indicating macroscopic tract atrophy, and reduced FDC, indicating combined pathology of fiber-specific axonal loss and tract atrophy. Our findings complement the existing body of imaging-based and histopathological research shedding light on the microscopical structural changes induced by CSVD which are already present at an early stage of the disease, such as axonal loss, demyelination and gliosis in WMH^57,58^. Compared to FBA metrics, changes of DTI metrics appeared in a less specific anatomical distribution indicating changes of white matter integrity throughout large sections of the white matter skeleton. These findings are in line with previous studies revealing an increased MD as well as a decreased FA in patients with CSVD^59–61^. Lacking histopathological correlates for validation, we can only speculate on the differing results. In TBSS, false positives may be responsible for our findings, having been shown to arise from method-inherent issues like considerable noise dependency and projection inaccuracies in regions of crossing fibres^53^. Similarly, false-negative findings by FBA have to be considered as a cause, potentially arising from the relatively low b-value (b = 1000 mm/s^2^) leading to insufficient suppression of the extraaxonal compartment^62^.

Looking at regions with significantly differing FBA-metrics between participants with low and high extent of WMH, we identify specific white matter tracts that were preferentially affected: We found reduced FD in the corpus callosum, inferior and superior longitudinal fasciculus indicating a loss of diverse white matter fibre populations. A reduced FC and FDC in the corpus callosum, corticospinal tract inferior and superior longitudinal fasciculus further indicated white matter atrophy in these tracts. As suggested by consistent results from all analyses, the corpus callosum specifically appeared to be affected by fibre-specific axonal loss and atrophy. In accordance with these results, previous evidence and our own DTI analysis show significantly reduced FA and increased MD in callosal regions accompanying CSVD, a pattern which is coherent with DTI metric alterations in WMH and surrounding NAWM^4,63^.

The pronounced involvement of long-range white matter tracts such as the superior longitudinal fasciculus to chronic vascular injury in CSVD appears plausible due to their hemisphere-centric position which is the region of highest WMH frequency and fixel alterations in our analysis. The axons of these long fascicles are typically densely packed and of pronounced myelination which makes them a critical target of blood supply owing to their high metabolic maintenance costs^64^. Their vulnerability may be caused by their distance from well-perfused pericortical areas rendering them reliant on the branches of small perforators. These small-caliber vessels, however, show critical pathology in CSVD and are therefore eponymous. Inter alia, they feature arteriolosclerosis, lipohyalinosis and fibrinoid necrosis which coincide with impaired blood–brain barrier function and vasoreactivity^65–68^. Taken together, our findings match previous evidence in this sample showing disturbed structural connectivity preferentially in long-range fibre tracts in participants with higher CSVD burden^28^. In summary, widespread white matter alterations can be detected even at an early disease stage. Since the progression of CSVD appears to be modifiable by pharmacological intervention and lifestyle changes—e.g., antihypertensive therapy and smoking cessation—early disease characterization might enable more effective disease management and thus prevention of symptom emergence^65^.

Several imaging and histopathological studies have suggested that microstructural alterations in CSVD spread across lesions detectable in T2-images^58,59,61,69,70^. On that basis, recent longitudinal evidence indicates that WMH are being preceded by subvisible alterations leading to the hypothesis that CSVD manifestations lie on an injury continuum with WMH marking regions of more advanced pathology^9,15,71,72^. Our findings are in line with this conception. We found that tracts identified as affected by our tract of interest analysis—the corpus callosum, corticospinal tract, inferior longitudinal fasciculus and superior longitudinal fasciculus—exhibited higher amounts of WMH load in the tract-wise assessment. In the whole-brain FBA, FDC changes consistently overlapped with regions of frequent WMH appearance.

Impairment of frontal-executive cognitive functions is one of the main clinical characteristics of vascular cognitive impairment—conditions for which CSVD is regarded as a key cause^73^. In our study, adjusted fixel- and voxel-wise linear models indicated an association between reduced FD (FBA) and increased MD (DTI) with longer processing times (determined by TMT-delta) in white matter tracts such as the superior longitudinal fasciculus, arcuate fasciculus and inferior longitudinal fasciculus. These findings are in line with the dependency of executive brain functions on large-scale brain networks integrating processes from remote brain regions^27^. Therefore, our results would support the relevance of long-range fibres for integrating distinct brain functions distributed throughout the neuronal anatomy. Previous investigations demonstrated an association of the superior longitudinal fasciculus’ integrity as detected by DTI metrics and cognitive function in CSVD^64,65^. We did not observe a statistically significant association between FA and TMT performance. A similar observation has been reported before^74^.

This study has several additional limitations. Due to the cross-sectional study design, this work captures only a snapshot of disease characteristics. Therefore, there is considerable potential to elucidate the progression of white matter alteration utilizing a longitudinal fixel framework^75,76^. The general limitations of our diffusion imaging-based methodology should be considered. Diffusion-to-axon mapping—meaning inferring white matter fibre configuration from DWI—requires lots of assumption making it an inherently complex endeavour to draw scientific conclusions from it. Specifically, reconstructions appear to be relevantly influenced by the decisions made in the acquisition protocol like amount of gradient directions, signal-to-noise-ratio and diffusion weighting^77^. In addition, DWI has a relatively low signal-to-noise ratio and is sensible for artifacts like susceptibility distortions and eddy currents and head motion^78,79^. These issues might complicate adequate reconstructions of fibre orientations which FBA relies on.

One rather specific limitation of our approach is the acquisition of diffusion-weighted images with a b-value of b = 1000 s/mm^2^. Initially chosen because of a high signal-to-noise ratio the low b-value might lead to insufficient suppression of the extra-axonal compartment complicating quantification of the fibre density which relies on signal of the intraaxonal compartment. We note that the recommended b-value for quantifying fibre density is 2000s/mm^2^ and previous work has shown that results can differ between acquisitions with different b-values^21,62^. Hence our results regarding fibre density should be interpreted with the necessary care. However, we also highlight that our results regarding the fibre density can be deemed pathophysiologically plausible and that previous FBA-based analyses with a b-value of 1000 s/mm^2^ also came to conclusive results^42,43^. Also, the effects of lower b-values should be attenuated by the application of single-shell 3-tissue constrained spherical deconvolution during processing in our analysis.

## Conclusion

Fixel-based analysis revealed white matter alterations in form of abnormal axonal microstructure and tract macrostructure in subjects with CSVD. Altered white matter microstructure extended into normal-appearing white matter involving inter- and intrahemispheric, long-distance white matter tracts such as the corpus callosum and superior longitudinal fasciculus. In an exploratory analysis, altered white matter microstructure in the superior longitudinal fasciculus was associated with worse executive cognitive functions.

## Supplementary Information

Supplementary Information.

## Data Availability

Anonymized data of the analysis not published within this article will be made available on reasonable request from any qualified investigator after evaluation of the request by the Steering Board of the HCHS.
