# Supplementary material for: Fixel based analysis of white matter alterations in early stage cerebral small vessel disease
Source: Sci Rep. 2022 Jan 28;12:1581. doi: 10.1038/s41598-022-05665-2 (PMC8799636; doi:10.1038/s41598-022-05665-2)
Supplement: Supplementary file 1 — Supplementary Information. [file 41598_2022_5665_MOESM1_ESM.pdf]

Fixel Based Analysis of White Matter Alterations in Early Stage Cerebral Small Vessel Disease

Marvin Petersen[a], Benedikt M. Frey[a], Carola Mayer[a], Simone Kühn[b], Jürgen Gallinat[b], Uta Hanning[c], Jens Fiehler[c], Katrin Borof[d], Annika Jagodzinski[d,e], Christian Gerloff[a], Götz Thomalla[a], Bastian Cheng[a]

[a] Department of Neurology, University Medical Center Hamburg-Eppendorf, Hamburg, Germany

[b] Department of Psychiatry and Psychotherapy, University Medical Center Hamburg-Eppendorf, Hamburg, Germany

[c] Department of Diagnostic and Interventional Neuroradiology, University Medical Center Hamburg-Eppendorf, Hamburg, Germany

[d] Epidemiological Study Center, University Medical Center Hamburg-Eppendorf, Hamburg, Germany

[e] Department of General and Interventional Cardiology, University Heart and Vascular Center, Hamburg, Germany

Corresponding author:
Marvin Petersen
mar.petersen@uke.de
Department of Neurology
University Medical Center Hamburg-Eppendorf
Martinistraße 52, 20246 Hamburg, Germany

Contents

Supplementary Figure 1. Clinical Fixel-based Analysis - TMT-A 3
Supplementary Figure 2. Clinical Fixel-based Analysis - TMT-B 4
Supplementary Figure 3. Tract of Interest Analysis – FD 5
Supplementary Table 1. Tract of Interest Analysis - FD 6
Supplementary Figure 4. Tract of Interest Analysis - Log FC 7
Supplementary Table 2. Tract of Interest Analysis - Log FC 8
Supplementary Table 3. Tract of Interest Analysis - FDC 9
Supplementary Table 4. Tract of Interest Analysis - WMH Load 10
Supplementary Text 1. Acknowledgement of HCHS committees 11

Supplementary Figure 1. Clinical Fixel-based Analysis - TMT-A

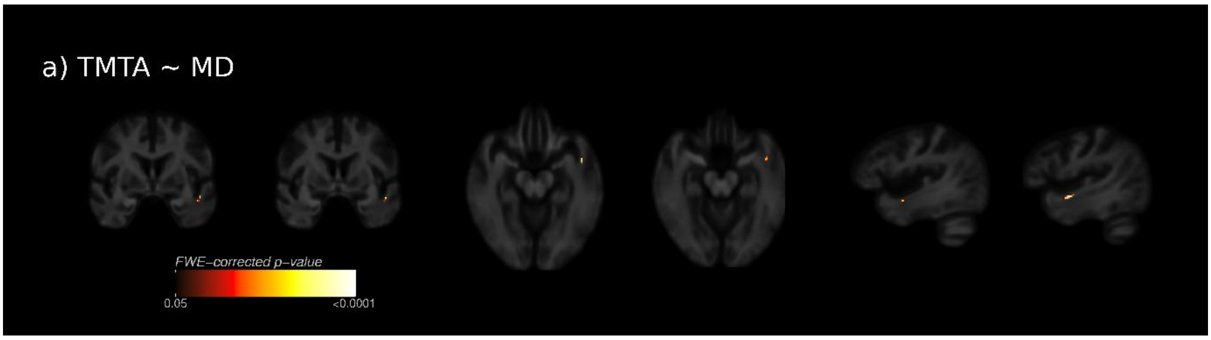

Linear relationship of TMT-A and diffusion metrics. a: TBSS skeleton voxels that show a significant linear relationship between the MD and the difference of TMT-A are displayed on the group template. There were no significant linear associations between TMT-A and FD, FC, FDC as well as FA respectively. Abbreviations: FA = fractional anisotropy, FD = fibre density, FC = fibre-bundle cross-section, FDC = Fibre density and cross-section, MD = mean diffusivity, TMT-A = trail making test a. Figure was produced using inkscape and mrview from MRtrix3 (https://www.mrtrix.org/).

Supplementary Figure 2. Clinical Fixel-based Analysis - TMT-B

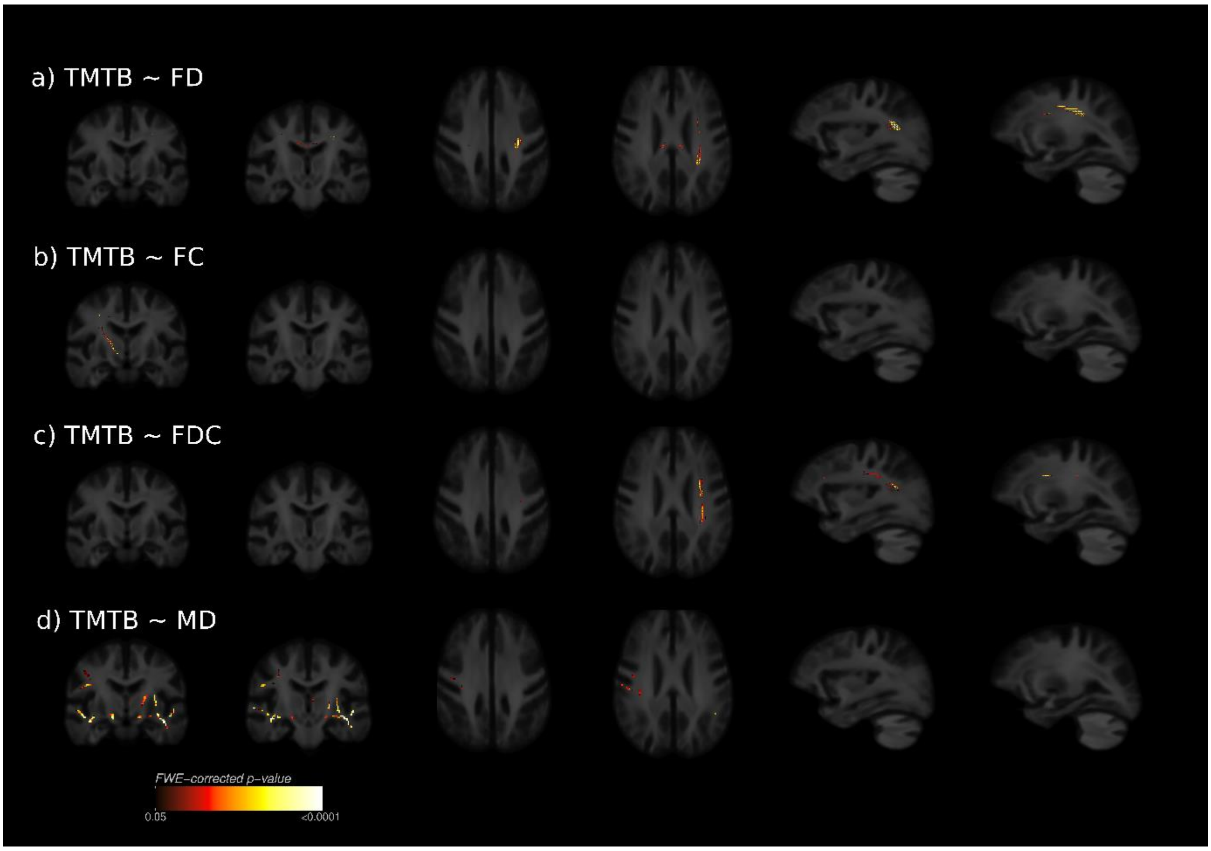

Linear relationship of TMT-B and diffusion metrics. a-c: Fixels exhibiting a significant linear relationship between TMT-B and FD (a), FC (b) and FDC (c) are displayed on the population template. d: TBSS skeleton voxels that show a significant linear relationship between the MD and TMT-B are displayed on the group template. There were no significant linear associations between TMT-B and FA. Abbreviations: FA = fractional anisotropy, FD = fibre density, FC = fibre-bundle cross-section, FDC = Fibre density and cross-section, MD = mean diffusivity, TMT-A = trail making test a. Figure was produced using inkscape and mrview from MRtrix3 (https://www.mrtrix.org/).

Supplementary Figure 3. Tract of Interest Analysis – FD

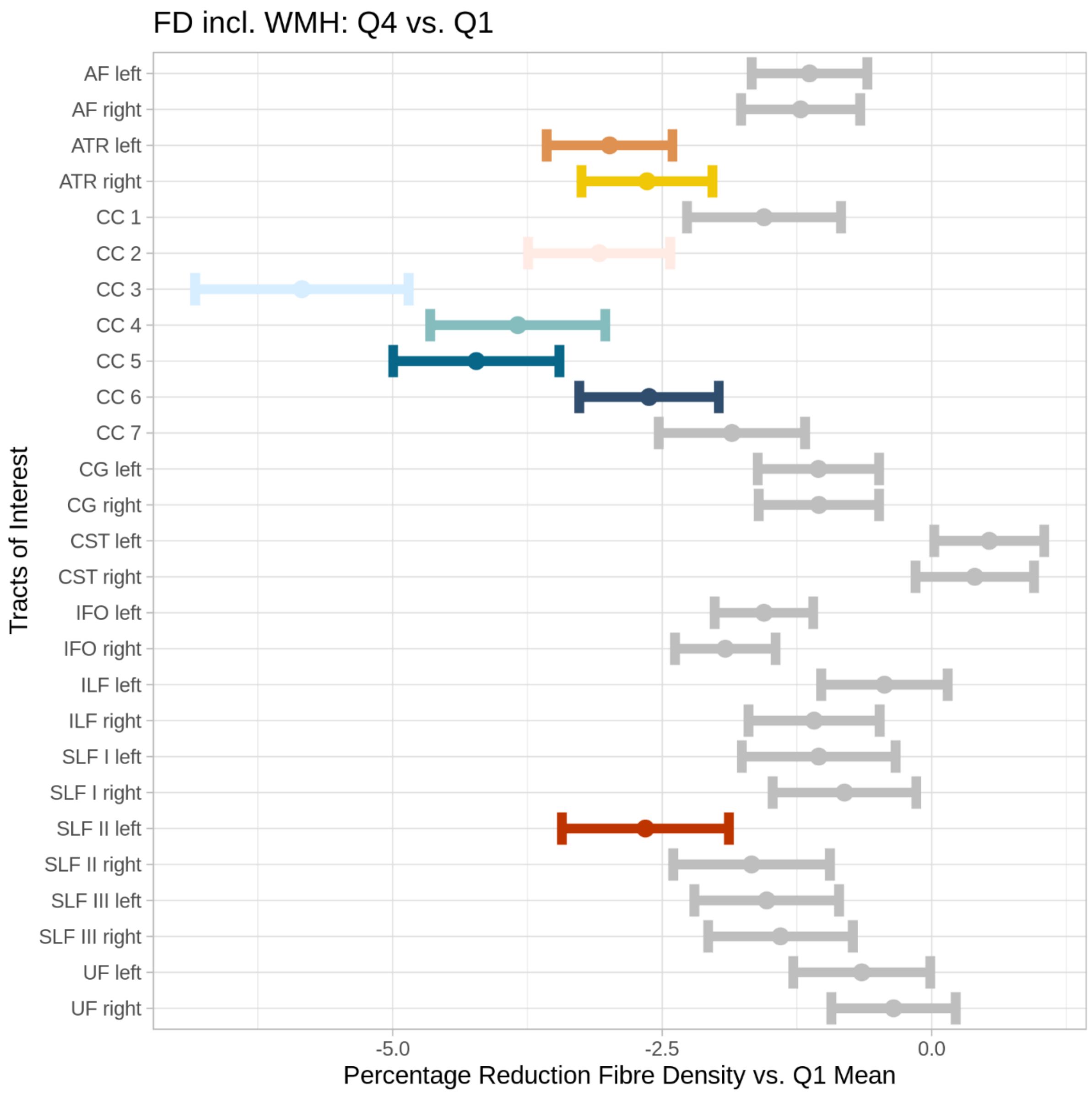

Supplementary Table 1. Tract of Interest Analysis - FD

variable	estimate	SE	df	z.ratio	p.value	p.value_bonf	p.value_report
AF left	0.543966	0.540429	Inf	1.006545	0.314154	60.63165	
AF right	0.626483	0.540429	Inf	1.159232	0.246362	47.5478	
ATR left	2.398624	0.540429	Inf	4.438367	9.06E-06	0.001749	***
ATR right	2.051635	0.540429	Inf	3.796304	0.000147	0.028346	*
CC 1	0.966129	0.540429	Inf	1.787706	0.073823	14.24792	
CC 2	2.495277	0.540429	Inf	4.617211	3.89E-06	0.000751	***
CC 3	5.252592	0.540429	Inf	9.719293	2.50E-22	4.82E-20	***
CC 4	3.250339	0.540429	Inf	6.014364	1.81E-09	3.49E-07	***
CC 5	3.635229	0.540429	Inf	6.726557	1.74E-11	3.35E-09	***
CC 6	2.032768	0.540429	Inf	3.761394	0.000169	0.032611	*
CC 7	1.263847	0.540429	Inf	2.338597	0.019356	3.735762	
CG left	0.461535	0.540429	Inf	0.854015	0.393096	75.86762	
CG right	0.457194	0.540429	Inf	0.845983	0.397562	76.72952	
CST left	-1.12356	0.540429	Inf	-2.07902	0.037615	7.259756	
CST right	-0.98904	0.540429	Inf	-1.83009	0.067236	12.9766	
IFO left	0.966709	0.540429	Inf	1.78878	0.07365	14.2145	
IFO right	1.325163	0.540429	Inf	2.452056	0.014204	2.741424	
ILF left	-0.15137	0.540429	Inf	-0.28009	0.779411	150.4264	
ILF right	0.501309	0.540429	Inf	0.927613	0.353608	68.24643	
SLF I left	0.45843	0.540429	Inf	0.848271	0.396287	76.48344	
SLF I right	0.219537	0.540429	Inf	0.406226	0.684576	132.1232	
SLF II left	2.066132	0.540429	Inf	3.823129	0.000132	0.025431	*
SLF II right	1.081536	0.540429	Inf	2.001253	0.045365	8.755474	
SLF III left	0.941011	0.540429	Inf	1.741227	0.081644	15.75724	
SLF III right	0.81337	0.540429	Inf	1.505043	0.132313	25.53639	
UF left	0.059378	0.540429	Inf	0.109872	0.912511	176.1146	
UF right	-0.23564	0.540429	Inf	-0.43602	0.662824	127.925	

Supplementary Figure 4. Tract of Interest Analysis - Log FC

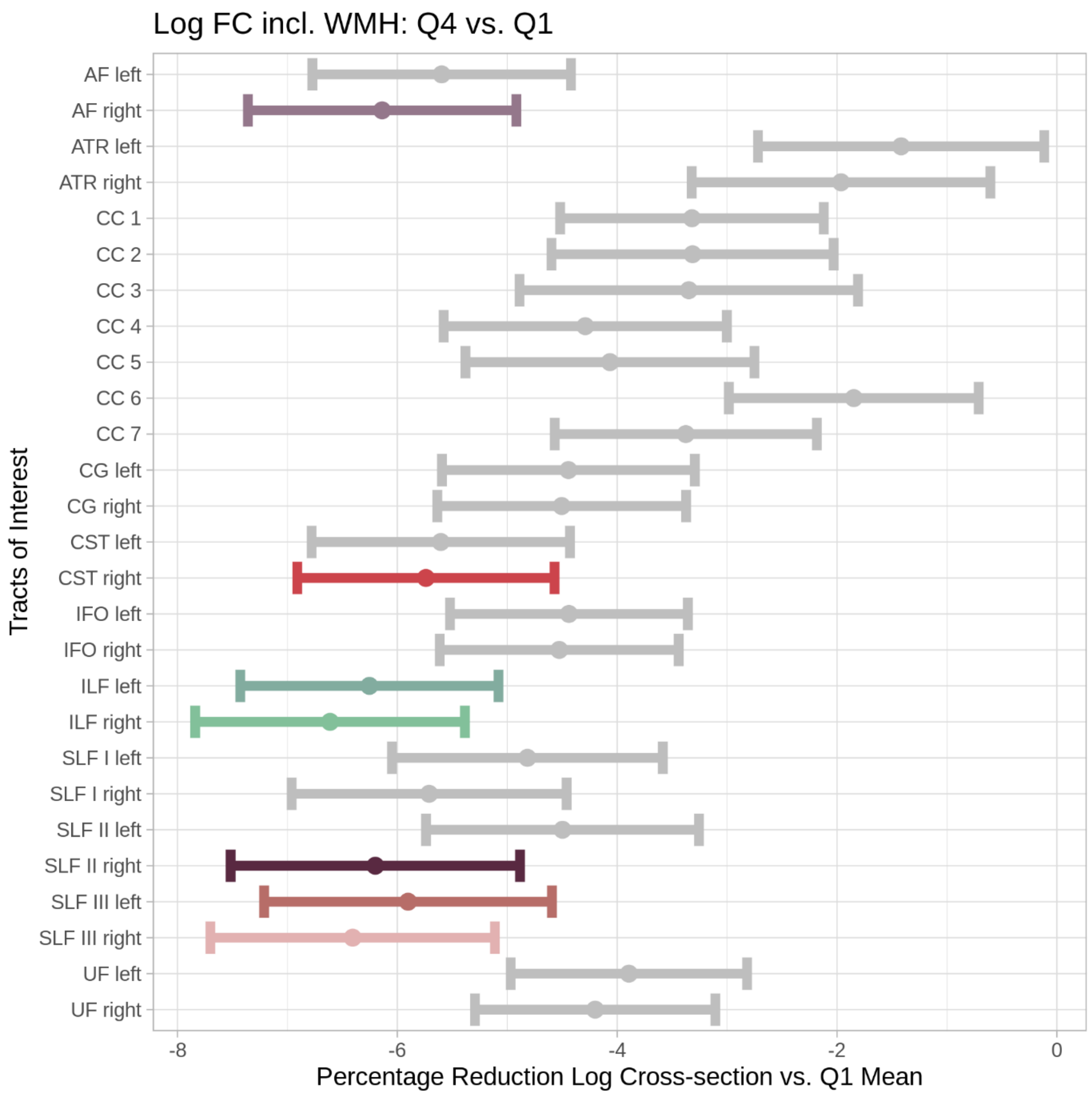

Supplementary Table 2. Tract of Interest Analysis - Log FC

variable	estimate	SE	df	z.ratio	p.value	p.value_bonf	p.value_report
AF left	2.239009	0.656361	Inf	3.411248	0.000647	0.124806	
AF right	2.754107	0.656361	Inf	4.196027	2.72E-05	0.005243	**
ATR left	-1.95487	0.656361	Inf	-2.97835	0.002898	0.559331	
ATR right	-1.46716	0.656361	Inf	-2.23529	0.025398	4.901885	
CC 1	-0.02825	0.656361	Inf	-0.04304	0.965672	186.3747	
CC 2	-0.05621	0.656361	Inf	-0.08563	0.931758	179.8292	
CC 3	-0.16474	0.656361	Inf	-0.25099	0.801821	154.7515	
CC 4	0.891606	0.656361	Inf	1.358408	0.174334	33.6465	
CC 5	0.671517	0.656361	Inf	1.023091	0.306265	59.10909	
CC 6	-1.49818	0.656361	Inf	-2.28256	0.022457	4.334119	
CC 7	0.056983	0.656361	Inf	0.086817	0.930817	179.6477	
CG left	1.075194	0.656361	Inf	1.638115	0.101398	19.56975	
CG right	1.146294	0.656361	Inf	1.74644	0.080735	15.58178	
CST left	2.30324	0.656361	Inf	3.509108	0.00045	0.086775	
CST right	2.434729	0.656361	Inf	3.709438	0.000208	0.04009	*
IFO left	1.180789	0.656361	Inf	1.798995	0.072019	13.89975	
IFO right	1.249182	0.656361	Inf	1.903195	0.057015	11.00391	
ILF left	2.935915	0.656361	Inf	4.473022	7.71E-06	0.001488	***
ILF right	3.270198	0.656361	Inf	4.982319	6.28E-07	0.000121	***
SLF I left	1.437109	0.656361	Inf	2.189512	0.02856	5.512018	
SLF I right	2.320105	0.656361	Inf	3.534802	0.000408	0.078759	
SLF II left	1.101491	0.656361	Inf	1.678179	0.093312	18.00924	
SLF II right	2.756502	0.656361	Inf	4.199676	2.67E-05	0.005159	**
SLF III left	2.431827	0.656361	Inf	3.705017	0.000211	0.040796	*
SLF III right	2.948346	0.656361	Inf	4.491961	7.06E-06	0.001362	***
UF left	0.63208	0.656361	Inf	0.963007	0.335544	64.75999	
UF right	0.927518	0.656361	Inf	1.413123	0.15762	30.42058	

Supplementary Table 3. Tract of Interest Analysis - FDC

variable	estimate	SE	df	z.ratio	p.value	p.value_bonf	p.value_report
AF left	3.030142	0.979556	Inf	3.093382	0.001979	0.381926	
AF right	3.459599	0.979556	Inf	3.531802	0.000413	0.079659	
ATR left	1.032915	0.979556	Inf	1.054472	0.291667	56.29172	
ATR right	1.013374	0.979556	Inf	1.034523	0.300892	58.07206	
CC 1	0.700766	0.979556	Inf	0.715391	0.474367	91.55291	
CC 2	2.503258	0.979556	Inf	2.555501	0.010603	2.046475	
CC 3	4.88473	0.979556	Inf	4.986675	6.14E-07	0.000119	***
CC 4	4.045677	0.979556	Inf	4.130111	3.63E-05	0.006998	**
CC 5	4.212842	0.979556	Inf	4.300764	1.70E-05	0.003285	***
CC 6	0.708356	0.979556	Inf	0.723139	0.469594	90.63173	
CC 7	0.722749	0.979556	Inf	0.737832	0.460616	88.89894	
CG left	1.50018	0.979556	Inf	1.531489	0.125649	24.25019	
CG right	1.409947	0.979556	Inf	1.439373	0.150045	28.95868	
CST left	0.916595	0.979556	Inf	0.935725	0.349415	67.43707	
CST right	1.50886	0.979556	Inf	1.540351	0.123475	23.83066	
IFO left	2.216209	0.979556	Inf	2.262462	0.023669	4.568093	
IFO right	2.646195	0.979556	Inf	2.701421	0.006904	1.332546	
ILF left	2.681057	0.979556	Inf	2.737011	0.0062	1.196605	
ILF right	3.526624	0.979556	Inf	3.600226	0.000318	0.061363	
SLF I left	1.747454	0.979556	Inf	1.783924	0.074436	14.36615	
SLF I right	2.312021	0.979556	Inf	2.360274	0.018261	3.524463	
SLF II left	3.161049	0.979556	Inf	3.227021	0.001251	0.241417	
SLF II right	3.792621	0.979556	Inf	3.871773	0.000108	0.020853	*
SLF III left	3.369122	0.979556	Inf	3.439436	0.000583	0.112505	
SLF III right	3.697238	0.979556	Inf	3.7744	0.00016	0.030956	*
UF left	0.56374	0.979556	Inf	0.575506	0.564949	109.0352	
UF right	0.485396	0.979556	Inf	0.495526	0.620229	119.7042	

Supplementary Table 4. Tract of Interest Analysis - WMH Load

variable	estimate	SE	df	z.ratio	p.value	p.value_bonf	p.value_report
AF left	-0.43911	0.055595	Inf	-7.89823	2.83E-15	5.46E-13	***
AF right	-0.53156	0.055595	Inf	-9.56121	1.16E-21	2.25E-19	***
ATR left	-1.37274	0.055595	Inf	-24.6916	1.32E-134	2.54E-132	***
ATR right	-1.41218	0.055595	Inf	-25.401	2.46E-142	4.74E-140	***
CC 1	-0.17884	0.055595	Inf	-3.21674	0.001297	0.250237	
CC 2	-0.82688	0.055595	Inf	-14.8731	4.93E-50	9.51E-48	***
CC 3	-1.71976	0.055595	Inf	-30.9334	4.24E-210	8.19E-208	***
CC 4	-1.36312	0.055595	Inf	-24.5186	9.36E-133	1.81E-130	***
CC 5	-0.7756	0.055595	Inf	-13.9508	3.11E-44	6.00E-42	***
CC 6	-0.73378	0.055595	Inf	-13.1986	8.94E-40	1.73E-37	***
CC 7	-1.14191	0.055595	Inf	-20.5397	9.51E-94	1.84E-91	***
CG left	-0.1426	0.055595	Inf	-2.565	0.010318	1.991309	
CG right	-0.15387	0.055595	Inf	-2.76767	0.005646	1.089662	
CST left	-0.76174	0.055595	Inf	-13.7014	9.95E-43	1.92E-40	***
CST right	-0.62387	0.055595	Inf	-11.2216	3.19E-29	6.16E-27	***
IFO left	-0.86322	0.055595	Inf	-15.5269	2.28E-54	4.40E-52	***
IFO right	-0.92681	0.055595	Inf	-16.6707	2.14E-62	4.13E-60	***
ILF left	-0.23568	0.055595	Inf	-4.23917	2.24E-05	0.00433	***
ILF right	-0.29771	0.055595	Inf	-5.35485	8.56E-08	1.65E-05	***
SLF I left	-0.34732	0.055595	Inf	-6.24734	4.17E-10	8.06E-08	***
SLF I right	-0.47967	0.055595	Inf	-8.6278	6.25E-18	1.21E-15	***
SLF II left	-0.86394	0.055595	Inf	-15.5397	1.87E-54	3.61E-52	***
SLF II right	-0.67982	0.055595	Inf	-12.2279	2.20E-34	4.26E-32	***
SLF III left	-0.60686	0.055595	Inf	-10.9157	9.70E-28	1.87E-25	***
SLF III right	-0.65438	0.055595	Inf	-11.7703	5.55E-32	1.07E-29	***
UF left	-0.42979	0.055595	Inf	-7.73068	1.07E-14	2.06E-12	***
UF right	-0.49925	0.055595	Inf	-8.98012	2.70E-19	5.22E-17	***

Supplementary Text 1. Acknowledgement of HCHS committees

Founding Board:

Adam, Gerhard

Blankenberg, Stefan

Koch-Gromus, Uwe

Gerloff, Christian

Jagodzinski, Annika

List of Investigators:

Adam, Gerhard

Aarabi, Ghazal

Augustin, Matthias

Behrendt, Christian

Beikler, Thomas

Betz, Christian

Blankenberg, Stefan

Bokemeyer, Carsten

Brassen, Stefanie

Brekenfeld, Caspar

Briken, Peer

Busch, Chia-Jung

Büchel, Christian

Debus, Eike Sebastian

Fiehler, Jens

Gallinat, Jürgen

Gellißen, Simone

Gerloff, Christian

Girdauskas, Evaldas

Gosau, Martin

Härter, Martin

Harth, Volker

Heydecke, Guido

Huber, Tobias

Jagodzinski, Annika

Johansen, Christoffer

Koch-Gromus, Uwe

Konnopka, Alexander

König, Hans-Helmut

Kromer, Robert

Kubisch, Christian

Kühn, Simone

Löwe, Bernd

Lund, Gunnar

Meyer, Christian

Nienhaus, Albert

Pantel, Klaus

Püschel, Klaus

Reichenspurner, Hermann,

Sauter, Guido

Scherer, Martin

Schnabel, Renate

Schulz, Holger

Smeets, Ralf

Spitzer, Martin S.

Terschüren, Claudia

Thomalla, Götz

von dem Knesebeck, Olaf

Waschki, Benjamin

Wegscheider, Karl

Zeller, Tanja

Zyriax, Birgit-Christiane

Steering Board:

Augustin, Matthias

Blankenberg, Stefan

Gallinat, Jürgen

Gerloff, Christian

Härter, Martin

Jagodzinski, Annika

Johansen, Christoffer

Koch-Gromus, Uwe

Sauter, Guido

Zeller, Tanja

Wegscheider, Karl

Betz, Christian/ Heydecke, Guido/ Gosau, Martin

Research consortium:

Aarabi, Ghazal

Andrees, Valerie

Behrendt, Christian Brassen, Stefanie

Brekenfeld, Caspar

Brünahl, Christian

Busch, Chia-Jung

Freitag, Janina

Gallinat, Jürgen

Gellißen, Susanne

Girdauskas, Evaldas

Heidemann, Christoph

Hussein, Yassin

Klein, Verena

Kofahl, Christopher

Kohlmann, Sebastian

Konnopka, Alexander

Kühn, Simone

Lühmann, Dagmar

Lund, Gunnar

Magnussen, Christina

Meyer, Christian

Nagel, Lina

Petersen, Elina

Scherschel, Katharina

Schiffner, Ulrich

Schnabel, Renate

Schulz, Holger

Seedorf, Udo

Smeets, Ralf

Terschüren, Claudia

Thomalla, Götz

Waschki, Benjamin

Zeller, Tanja

Zyriax, Birgit-Christiane
